# Development and Validation of an Automated Step Ergometer

**DOI:** 10.2478/hukin-2014-0096

**Published:** 2014-11-12

**Authors:** Maria do Socorro C. de Sousa, Rodrigo R. Aniceto, Gabriel R. Neto, Ravi C. T. de Araújo, Juliana B. C. de Sousa, José A. D. Costa, Idico L. Pellegrinotti

**Affiliations:** 1 Kinanthropometry and Human Performance Laboratory, Federal University of Paraíba, João Pessoa, Brazil.; 2 Physical Education Post-graduate Associate Program, Federal University of Paraíba, João Pessoa, Brasil.; 3 Laboratory of Kinesiology and Biomechanics, Integrated Colleges of Patos, Patos, Brasil.; 4 Physical Education Post-graduate Program, Federal University of Rio de Janeiro, Rio de Janeiro, Brasil.; 5 Federal Institute of Education, Science and Technology of Paraiba, João Pessoa, Brazil.; 6 Methodist University of Piracicaba, Piracicaba, Brazil.

**Keywords:** step test, ergometer, heart rate, cardiorespiratory fitness, oxygen uptake

## Abstract

Laboratory ergometers have high costs, becoming inaccessible for most of the population, hence, it is imperative to develop affordable devices making evaluations like cardiorespiratory fitness feasible and easier. The objective of this study was to develop and validate an Automated Step Ergometer (ASE), adjusted according to the height of the subject, for predicting VO2max through a progressive test. The development process was comprised by three steps, the theoretical part, the prototype assembly and further validation. The ASE consists in an elevating platform that makes the step at a higher or lower level as required for testing. The ASE validation was obtained by comparing the values of predicted VO2max (equation) and direct gas analysis on the prototype and on a, treadmill. For the validation process 167 subjects with average age of 31.24 ± 14.38 years, of both genders and different degrees of cardiorespiratory fitness, were randomized and divided by gender and training condition, into untrained (n=106), active (n=24) and trained (n=37) subjects. Each participant performed a progressive test on which the ASE started at the same height (20 cm) for all. Then, according to the subject’s height, it varied to a maximum of 45 cm. Time in each stage and rhythm was chosen in accordance with training condition from lowest to highest (60–180 s; 116–160 bpm, respectively). Data was compared with the student’s t test and ANOVA; correlations were tested with Pearson’s r. The value of α was set at 0.05. No differences were found between the predicted VO2max and the direct gas analysis VO2max, nor between the ASE and treadmill VO2max (p= 0.365) with high correlation between ergometers (r= 0.974). The values for repeatability, reproducibility, and reliability of male and female groups measures were, respectively, 4.08 and 5.02; 0.50 and 1.11; 4.11 and 5.15. The values of internal consistency (Cronbach’s alpha) among measures were all >0.90. It was verified that the ASE prototype was appropriate for a step test, provided valid measures of VO2max and could therefore, be used as an ergometer to measure cardiorespiratory fitness.

## Introduction

Nowadays, there are several methods to assess cardiorespiratory fitness in humans, ranging from non-specific field tests (Nakhostin-Roohi and Niknam, 2008; Weiglein et al., 2011) to the more specific ones where ergometers as a treadmill (Nemeth et al., 2009), bicycle (Kjelkenes and Thorsen, 2010) or steps (Hansen et al., 2011) are often used. In addition, there are some which are more specific to assess subjects with specific needs such as rowing ergometers (Kendall et al., 2011), wheelchair (Bernasconi et al., 2007) or arm ergometers (Laskin et al., 2004). There are several tests that can be performed on each of those aforementioned ergometers and several variables that can be assessed also, being the maximum oxygen uptake (VO_2max_) one of them. The VO_2max_ is commonly used for the assessment of human performance, namely aerobic (Lochte et al., 2009) being in general interpreted as an index of cardiorespiratory fitness. This kind of evaluations by means of ergometers is not only used in sports (Rankovic et al., 2010), but also in different occupational activities (Manero and Manero, 1991). In either case, this assessment allows a more accurate exercise prescription when looking for significant benefits with regard to fitness and health (Anton et al., 2011). Furthermore VO_2max_ is a useful parameter to assess the effects of physical training on the cardiorespiratory system (Hansen et al., 2011). Such tests are sometimes based on the linear relationship between the heart rate and oxygen uptake (Wicks et al., 2011), typically during submaximal steady-state exercise tests.

Unfortunately, modern ergometers such as a treadmill, cycle and rowing ergometers have high costs, becoming, together with the requirements of specific facilities for their installation, inaccessible for most of the population (Balderrama et al., 2010). In addition, these specialized apparatuses (e.g. treadmill) are highly technical to operate. Moreover, this kind of laboratory evaluations is less ecological than the one performed in more specific conditions. On the other hand step ergometers are simple and less expensive devices.

Previous studies that included the subject’s height in a step test protocol (Francis and Brasher, 1992; Ashley et al., 1997) have not presented increments with proper duration to prevent overload, thus limiting the heart rate assessment accuracy and hindering VO_2max_ prediction.

In order to address such drawbacks, we sought to develop an automated equipment with test, measurement and evaluation functions of cardiorespiratory fitness (VO_2_, ml/kg/min^−1^) in subjects of all ages (adolescents, adults and elderly), through ascend and descent movements (with continuous steps), rhythmically (metronome) and incrementally according to the subject’s height and physical fitness level.

The Automated Step Ergometer (ASE) is a simple, economical and efficient equipment combining small dimensions and portability, allowing to evaluate a large number of subjects in short time. Moreover, the ASE allows testing several people simultaneously.

Thus, the purpose of this study was to develop and validate the ASE as a predictor of VO_2max_ assessed in a typical treadmill progressive test.

## Material and Methods

### Participants

For the validation process 167 subject with average age of 31.24 ± 14.38 years, of both genders and different degrees of cardiorespiratory fitness were chosen and then randomized and divided by gender (men and woman), respectively, into untrained (n = 41 and n = 65), active (n = 12 and n = 12) and trained (n = 28 and n = 9) individuals. Each subject was informed of all tests procedures and gave their written informed consent to participate. The study was approved by the Ethics Committee of the University Federal of Paraiba (# 203/10).

### Development and Validation of the Ergometer

This is a descriptive study aiming to describe the development process of the ASE prototype for the prediction of VO_2max_ in three steps: the theoretical, the development and finally the validation of the prototype. ASE development was based on a step test with 6 progressive effort levels, justified by the need of load increments in each level of effort in order to assess cardiorespiratory fitness. ASE elevates itself along the test, according to the height of the subjects being tested.

### Theoretical Basis for the Construction of Automated Step Ergometer

In exercise physiology, particularly in laboratory tests, VO_2max_ is one of the most valid measures of aerobic power (Machado et al., 2002), thus widely used in the assessment of cardiorespiratory fitness of athletes, patients and workers. In this way, by choosing a given test and ergometer, the evaluator should take into account three factors: validity, reliability and sensitivity (Currell and Jeukendrup, 2008). Moreover, it should follow some psychometric quality criteria as objectivity, standardization, application viability, discrimination techniques and feedback for individuals.

The most reliable devices for the assessment of aerobic power are those that measure directly, through a mask placed on the face of the subject, the amount of gas exchanges. This direct gas analysis requires devices with several sensors and sophisticated software capable of measuring the oxygen uptake and carbon dioxide. Nonetheless, the required procedures make the use of such equipment restricted to laboratories at major research centers due to high cost for acquisition and maintenance. Even if this kind of equipment is in most cases unavailable for the majority of exercise professionals, still there are several valid methods used in determining the cardiorespiratory fitness which may be used frequently (treadmill, bicycle and step ergometers). All of these tests were validated based on strong correlations between the oxygen uptake and the heart rate. Furthermore, the VO_2max_ prediction tests can be applied either in maximum or sub-maximum efforts. Unlike maximum efforts (Myers et al., 2009), submaximal tests are usually safer and faster, because there is no need for medical supervision (Noonan and Dean, 2000). Step ergometers have been used in physical tests for decades, being the exercise characterized by ascending and descending from a step (or bench) on a determined pace for a few minutes. The VO_2max_ is estimated by means of regression equations between the heart rate and time (Mahon et al., 2010). However, some extremely important points should be considered for determining the cardiorespiratory fitness when using the step, for instance the height of the step (in relation to the height of the individual being tested), the execution speed (according to the degree of cardiorespiratory fitness) and progressive overload.

### Procedures and Protocols

Considering the aforementioned aspects, a step test protocol was developed based on the height of the subject in order to determine cardiorespiratory fitness. Starting with the right lower limb, with the foot resting on the surface of the step, the angle of 90° was established by bending of the hip and knee. In this way, six possible groups of heights were developed (Table 1), each one with three stages increments. The 1^st^ stage pertained to the minimum height of 20 cm. The 2^nd^ and 3^rd^ stages varied according to the height of the subject. The duration of the stages was established according to the fitness level of the subjects (untrained, active and trained), being respectively, 1^st^ stage (60 s for all individuals), 2^nd^ stage (60 s, 60 s and 120 s) and 3^rd^ stage (60 s, 120 s and 180 s). The change from one stage to the upcoming occurred within 10 s from the previous one.

The speed of movements was classified according to gender and cardiorespiratory fitness of the subjects (untrained, active, and trained) and an adequate range of beats/min was established and controlled by a metronome (for males 120 bpm, 144 bpm, and 160 bpm; and females 116 bpm, 132 bpm, and 152 bpm, respectively), being each four beats a complete cycle of ascent-ascent-descent-descent. Every stage needed height adjustment during test time, then the ASE allowed better and faster applicability, without heart rate measure losses caused by any interruptions to adjustments during the evaluation.

Before performing the step test all subjects were instructed to be adequately hydrated; to abstain from caffeine in the previous 4 hours; to abstain from alcohol and any physical activity 24 hours before testing. All guidelines were provided to subjects before and during the test. Familiarization was performed on the step for 15 s with the metronome to control the ascent and descent rhythm. Afterwards, the progressive effort test started with cycles of ascent-ascent-descent-descent, without alternating the legs and with full knee extension.

The criteria used to finalize the test were as follows: maximum heart rate within 10 beats of the maximum predicted for age; respiratory exchange rate higher than 1.1; and rating of perceived exertion above 18 of the Borg scale. The direct analysis of oxygen uptake was obtained by a metabolic analyzer AeroSport TEEM 100, during both the ASE and treadmill tests (Bruce, 1956). The values of oxygen uptake were recorded throughout the exercise, but only the end of 180, 240 and 360 s were considered for further analysis for untrained, trained and active subjects respectively.

### Statistical Analysis

Data normality and homogeneity were confirmed by the Kolmogorov-Smirnov and Levene tests, respectively. The Student *t* test was used to determine the differences between predicted and measured data. Concerning different ergometer tests, ANOVA and Pearson correlation (r) were used. Overall internal consistency was measured by the index of quality and measures of reliability (Cronbach’s alpha). The predicted VO_2max_ for the ASE was obtained through a regression equation, utilizing the following variables: age, height, body weight, gender, total test time, heart rate, step height, fitness level, and beats/minute. Data is presented as means and standard deviation and the adopted α value equaled 0.05. All statistical analyses were performed by means of the SPSS 16.0.

## Results

### Development of the Ergometer

As there was advancement in the lower stages of the protocol, it was required that the ASE platform raised to the pre-established values (Table 1). In this way the equipment could be operated remotely by a trained evaluator who selected a direction (ascent or descent) holding the green button (Picture 1 - C), making it possible to raise the platform to change stages without interrupting for manual adjustments.

The ergometer support structure (Picture 2) was assembled with the use of steel profiles in “U” format (SPU), aiming weight reduction. In the four ends of the steel structure that provided ground support, some cubes of wood coated with anti-slip rubber (EVA) were placed between the SPU (Table 2), allowing for greater stability during the tests. Columns of the same material with the same specifications (SPU), perpendicular to the base, allowed the support of the threaded cylindrical shank (TCS), for elevating the platform. The TCSs were turned in the bottom edge and placed in lubricated bronze bearings of two inches of diameter (Ø-2″). Bronze bearings were chosen because of their greater resistance to usage, since the applied forces were vertical and not horizontal. The TCSs passed through between the nuts of an inch and a half and had at its lower end a spur gear.

The elevating platform of the ASE consisted of a steel frame on “L” profile (SPL) in its laterals being its surface in pine wood coated with a non-slip tape. For the stabilization of the anterior-posterior movements of the platform during the ascent of the individuals, two steel brackets of 0.5 inches were placed in each column. The motor located in the base structure of the equipment had its axle coupled to a reduction gear box, which in turn held in its axis a cog wheel with 10 cm diameter (Ø - 10 cm) which transmitted through a chain of simple rollers with 150 cm, stretched and adjusted by two nylon rollers positioned on the side of the engine.

The force produced by the motor reached the chain rings fixed to the lower end of the TCs causing them to rotate to the left at the same speed, so that the nuts positioned on the columns moved in accordance with the received command (ascending or descending), allowing the vertical displacement of the platform from 20 cm to 50 cm from the floor (Picture 3).

The first transmission system functioned with a 0.25 horse power (HP) engine, being replaced afterwards by one with 2.0 HP. Even being a motor of low RPM, it has become necessary to reduce them to adjust the control of the ascent speed of the platform, thus increasing the engine power. The new transmission had 1820 revolutions per minute (RPM) and was reduced to 91 rpm, by a proportion of about 20: 1. The engine, metronome (Picture 1B) and control panel (Picture 1A) were powered by an electric current of 220 V.

The position selector located on the remote control (Picture 1C) indicated in which direction in the vertical plane the platform should move. The level of the height reached by the platform was limited by switches located at the top and bottom of the columns (limiting the displacement within 20–50 cm), so when the platform reached the upper or lower limit, the trigger switched inhibiting the electrical current to pass and thereby stabilizing the platform.

### Validation of Automated Step Ergometer

Cardiorespiratory fitness in the ASE was obtained through the following predictive equation:

$$\begin{array}{c} {\mathrm{VO}}_{2\max}=-93.402-[0.0548\;\times (\mathrm{age})]-[0.152\;\times \;(\mathrm{height})]-\\ {}[0.0874\;\times \;(\mathrm{weight})]-[0.568\times (\mathrm{gender})]+[0.05996\;\times \;\\ (\mathrm{time})]+[0.0118\;\times \;(\mathrm{final}\;\mathrm{HR})]+[0.798\;\times (\mathrm{step}\;\mathrm{height})]-\\ \left[16.221\;\times \;(\mathrm{fitness})]+[1.095\;\times \;(\mathrm{bpm}\;\mathrm{by}\;\mathrm{metronome})\right] \end{array}$$

where: age (in years); height (in centimeters); weight (in kilograms); gender (male/female = 0/1); time (total testing time in seconds); final HR (heart rate at the end of the test); step height (final height reached in the step); fitness (untrained/active/trained =1/2/3); bpm (beats per minute - untrained females = 116; active females = 132; trained females = 152; untrained males = 120; active males = 144; trained males = 160).

**Figure 1:**
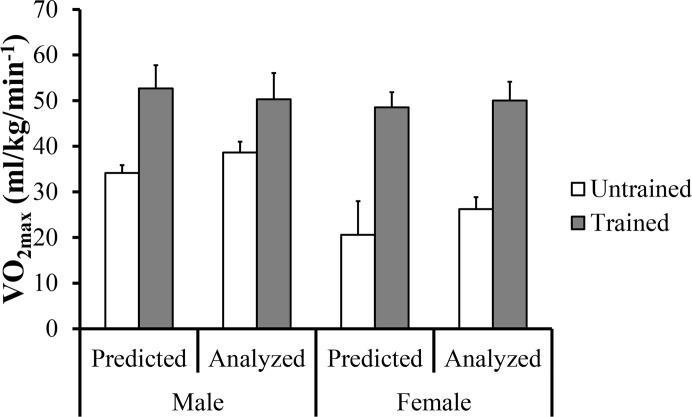
Values (mean and standard deviation) of predicted maximum oxygen uptake (VO_2max_) and analyzed by direct gas evaluation at the ASE.

Regarding the predicted value of VO_2_ obtained by the equation and the direct gas analysis evaluation on the ASE (Figure 1), no significant differences were found between the two test conditions.

Analyzing the values of VO_2_ (ml/kg/min^−1^; *p*= 0.365; *r*= 0.974), energy expenditure (kcal/min^−1^; *p*= 0.730; *r*= 0.997), metabolic equivalent (MET; *p*= 0.365; *r*= 0.746), heart rate (bpm; *p*= 1.095; *r*= 0.905) and the respiratory exchange rate (RER; *p*= 1.461; 0.466) we found no significant differences when comparing the exercise protocol applied on the ASE with the one on the treadmill. Additionally, a moderate to high correlation was observed between the ergometers.

**Figure 2:**
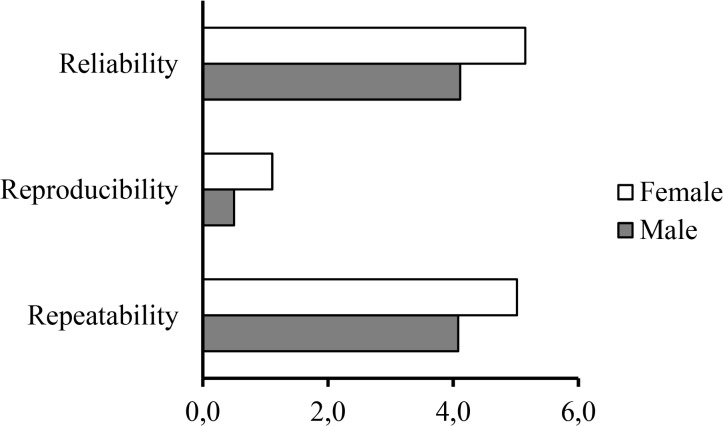
Measures quality (reliability, reproducibility and repeatability) of VO_2max_

The progressive test was submitted to an analysis of quality of measures looking to assess repeatability, reproducibility and reliability (Figure 2). Then an experiment was conducted using the same prediction equation with three evaluators performing two measurements in each experiment Accuracy was based on Cronbach’s alpha, followed by the Split Half method (method of halves) (Table 3). The evaluation of VO_2max_ through the equation on the ASE presented good quality regarding external validity stated by Cronbach’s alpha values of 0.90 and above. Moreover, it can be stated that in case of the two measures 98% of the variability was due to true variance and only 2% was attributed to error demonstrating a high degree of confidence by the instrument.

The quality of the measurements was determined through repeatability which evaluated the tolerance index of each evaluation of 2 measures of VO_2max_ among the three evaluators (A, B and C) (Table 3). According to the values obtained in the test, no corrective action was necessary in the prediction process of VO_2max_.

## Discussion

The objective of the present study was to develop and validate an Automated Step Ergometer for prediction of VO_2max_ based on a concurrent test.

As main results of this study, it was found that the ASE had the capacity to estimate VO_2max_ throughout a prediction equation, and that the step test adjusted according to individual height and degree of physical fitness showed similarity with a treadmill test. Thus, based on the predicted and analyzed VO_2max_, it is possible to advocate that the ASE is a valid instrument and provides accurate estimates of cardiorespiratory fitness in subjects aged 13–69 years.

The objective of this study was not an unprecedented attempt to predict VO_2max_ with indirect testing. For instance, Kline et al. (1987) using the one-mile test, created a predictive equation, and their results indicated that this walk test provided a valid assessment to estimate sub maximal VO_2max_. Therefore, in our study, we tried to validate the ASE comparing the predicted values with the values obtained by direct measures. Using a cycle ergometer, Siconolfi et al. (1982) experimented to estimate VO_2max_ in comparison with direct measures, concluding that there was no significant differences between both forms of analyzing VO_2max_, further presenting an index correction of 0.92 and 0.93 between the age groups. In the present study, there was no need to apply corrective actions regarding VO_2max_ prediction. Presenting a methodology similar to ours, Ingham et al. (2013) analyzed VO_2max_ variables between step-wise (*r*= 0.90) and ramp-wise (*r*= 0.88) in relation to 2000-m rowing ergometer performance, referring to a strong association between both protocols. In our study both protocols presented a high correlation (*r*= 0.974). Again, Siconolfi et al. (1985) observed that a step with 25.4 cm height provided accurate estimates of VO_2max_ and was safe and suitable for in-the-home assessment of fitness for people aged 19–70 years. In the present study, the step height ranged from 20 to 45 cm, depending on individual height and physical fitness. Regarding age, the participants of this study were aged 13 to 69 years. From the same perspective Francis and Brasher (1992) showed that the single-stage height adjusted step test provided an effective predictor of VO_2max_ in males and could be used when more complex methods of clinical testing were unavailable or not feasible. From our point of view, in this kind of testing approaches, one size does not fit all, since the effort that a taller subject has, comparing with a lower one, to accomplish the same step test is different. Moreover, one should consider the subjects’ individual physical fitness. Those variables were considered in the development of the ASE even though the study of Ashley et al. (1997) referred that step tests based on subjects’ stature did not predict cardiorespiratory fitness more accurately than those using a standardized bench height. Probably those results could be explained because of the fact that authors applied the maximum effort test and not the reaching steady-state, which promoted loss of linearity between the heart rate and oxygen uptake. For our study, each stage had a particular time and rhythm was controlled by a metronome, getting subjects to achieve levels of effort compatible with a steady-state. It is well known that the values obtained in VO_2max_ evaluations can be influenced by methodological issues (Astorino et al., 2005), also depending on the adaptation to training (Millet et al., 2009) and on genetic inheritance (Gaskill et al., 2001).

Although the present study meets the principle of variability of data in the cross-sectional design, it presents some limitations regarding the sample used. This step test on the ASE needs be applied in other populations such as children and patients with health conditions (cardiac, hypertensive, diabetic, obese). The ASE prototype, developed as any other equipment with mechanical and electrical items, also has operational limitations that may occur, yet simple to deal with. Among these we can mention the noise caused by the platform surface displacement and its mechanical screw which needs constant lubrication. Additionally, the dimensions may be reduced without safety prejudice regarding foot support of the evaluated individual.

Based on the analysis of the variables of this study, we can consider that the ASE was able to provide measurement of cardiorespiratory fitness, namely VO_2max_, in subjects with different cardiorespiratory fitness. In this way, the ASE and the suggested protocol is appropriate and can be used in predicting cardiorespiratory fitness (VO_2max_). Being easy to apply and to transport, the ASE can be an option for different environments such as schools, gyms, clubs, hospitals, businesses and others facilities, where the main purpose is the cardiorespiratory fitness. Therefore, within a sub-maximum effort perspective, the ASE use may represent a practicable alternative for a laboratory ergometer as it is low cost, enables easy access, has an easy protocol and operation management even in several individuals, and it has no risks to individuals’ health.

## Practical Implication

This study reports the scientific process of development and validation of an ASE, designed with the objective of testing, measuring and evaluating the cardiorespiratory fitness by predicting VO_2max_. Scientifically, it was compared with other ergometers and showed high correlations among their measurements with low risk to health. Through a relatively short time assessment (3–6 min) with an evaluation protocol of simple administration (simulates the natural movement of the step up and down) and the safety provided by the accommodation position during movement, it allows to measure the heart rate during both exercise and recovery. Thus, the ASE is appropriate in both clinical practices as well as in different environments; with a single subject or in groups; justified for professionals who seek equipment with reliable measures, and easiness in the test administration. This ergometer is efficiently and economically capable of assisting individuals of different ages (adolescents, adults and elderly) with different fitness levels (trained and untrained). Furthermore, it can be utilized in special conditions in relation to health status (cardiac, diabetic, obese, hypertensive), as it uses the heart rate and submaximal efforts.

## Figures and Tables

**Picture 1:**
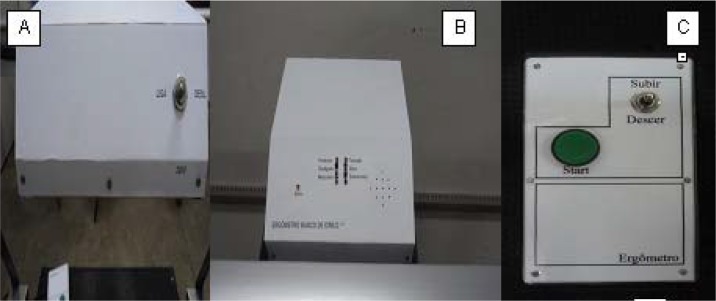
**The** Automated Step Ergometer control panel, metronome (A and B) and elevation control (C)

**Picture 2:**
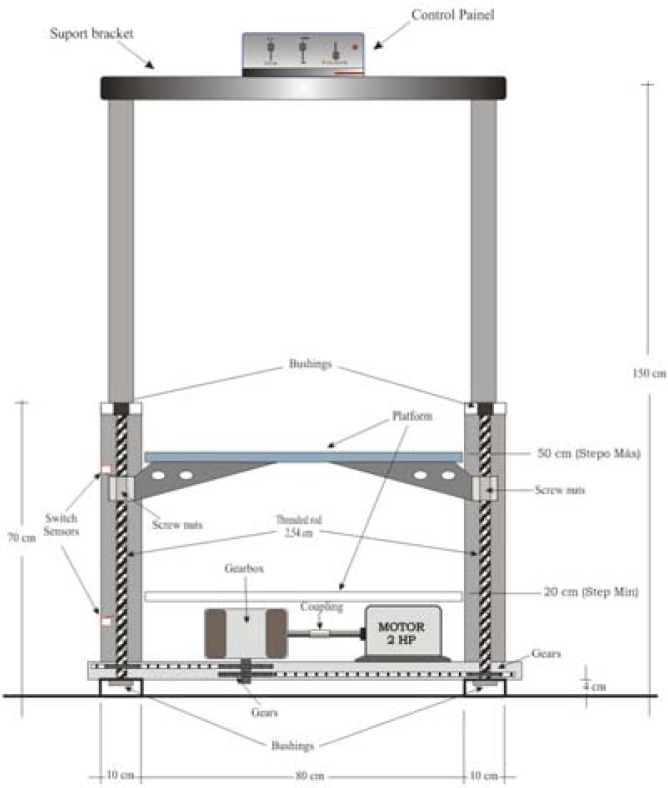
Automated Step Ergometer structure from the front view.

**Picture 3:**
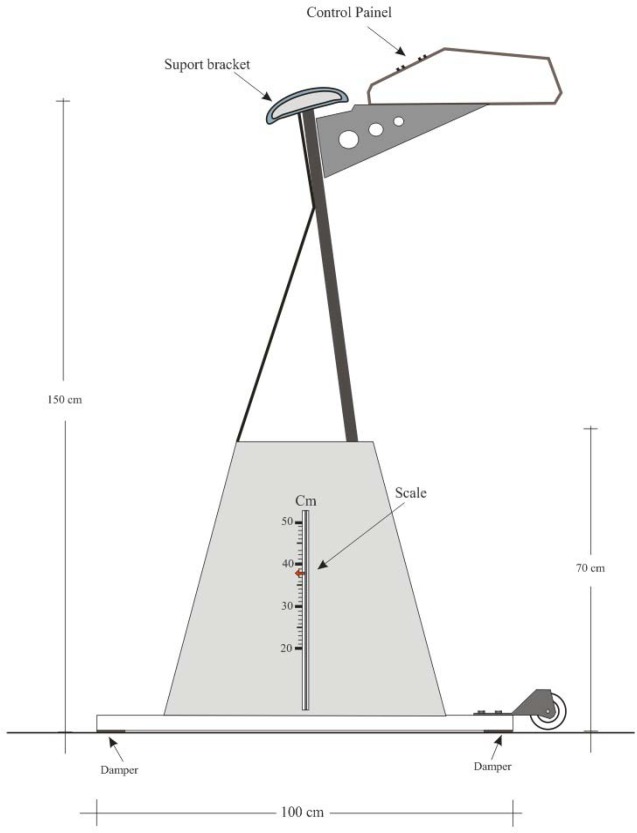
Automated Step Ergometer structure from the side view.

**Table 1:** Number of stages (heights) based on the subject height for the step test

*Body height (cm)*	*Stage 1 (cm)*	*Stage 2 (cm)*	*Stage 3 (cm)*
< 151.9	20	26	32
152.0 – 161.9	20	27	34
162.0 – 171.9	20	29	38
172.0 – 181.9	20	30	40
182.0 – 191.9	20	31	42
192.0 forward	20	32.5	45

cm: centimeters

**Table 2:** Specification and materials used to build the ergometer

*Material*	*Specifications*
Steel profile in “U” (SPU)	1.5″ × 3.0 × 1.5″
Steel profile in “L”(SPL)	2″ × 2″
Steel profile in “L”(SPL)	1″ × 1″
Profile of Steel plate	½″
Profile of Steel plate	2″(1/8″)
Profile of Steel plate	3″(1/8″)
Threaded cylindrical shank (TCS)	1 ½″
The threaded rod nuts	1 ½″
Cylindrical bronze bearings	Ø-2 ″
Low rotation Motor WEG c/reversal	220 Vac-2.0 HP
Speed reduction Box	20:1
Sprocket	Ø − 10 cm
Simple roller chain	1.5 m
Electric key S2	3 poles × 2 positions
Electric key S1 (Push Botton)	1 pole × 2 positions
Wires	Double – 12 AWG
Electric welding electrodes	120 A
Aluminium plate (Fairing)	N^º^ 18
Rectangular aluminium profile (grip)	2″ × 5″ Matte
Aluminium profile – handrail (Handle support)	Oval
Bronze bearings	3″ × 1″
Pine wood (platform)	1.0 m × 0 m × 0 m
Ethylene vinyl acetate (EVA) with friction coating	-

″: Inches; Vac: voltage electric current; Ø: diameter; HP: horse power; cm: centimeter; A: ampere; m: meter.

**Table 3:** Cronbach’s alpha of VO_2max_ measures (ml/kg/min^−1^) obtained by three evaluators (A, B and C) with two measurements each.

*Evaluator*	*Correlations (r)*	*Reliability (if the item is deleted)*	*Alpha Cronbach*
1^st^ Measurement			
A	0.9998	0.9993	0.9995
B	0.9991	0.9991	
C	0.9987	0.9994	
2^nd^ Measurement			
A	0.9984	0.9994	0.9995
B	0.9989	0.9991	
C	0.9990	0.9990	
1^st^ and 2^nd^ Measurements			
A	0.9991	0.9997	0.9998
B	0.9992	0.9997	
C	0.9992	0.9997	
A	0.9990	0.9997	
B	0.9990	0.9997	
C	0.9993	0.9997	

VO_2max_: maximum oxygen uptake.
